# A giant myxoma originating from the aortic valve causing severe left ventricular tract obstruction: a case report and literature review

**DOI:** 10.1186/s12957-015-0575-9

**Published:** 2015-04-16

**Authors:** Edvin Prifti, Fadil Ademaj, Efrosina Kajo, Arben Baboci

**Affiliations:** Division of Cardiac Surgery, University Hospital Center of Tirana, Tirana, Albania; Division of Cardiology, Regional Hospital of Gjakova, Rr. Prizren, Gjakova, Kosovo

**Keywords:** Aortic valve, Myxoma, Left ventricular tract obstruction

## Abstract

**Introduction:**

The left ventricular localization of a myxoma is very rare, usually arising from the interventricular septum close to the left ventricular outflow tract, the mitral valve, the ventricular wall and extremely rarely the aortic valve.

**Case presentation:**

A 13-year-old male was admitted due to dyspnea and angina. Transesophageal echocardiography revealed left ventricular outflow tract obstruction with a mean gradient of 58 mmHg, and a mobile mass measuring 65 × 25 mm originating from the ventricular surface of the aortic valve was identified. The patient underwent urgent surgical excision and aortic valve replacement. Histopathological examination of the mass confirmed the diagnosis of a myxoma.

**Conclusion:**

In conclusion, a myxoma originating from the aortic valve remains a very rare localization. Total resection associated with aortic valve replacement seems to offer an excellent outcome.

## Background

Most cardiac myxomas are located in the left atrium, attached to the interatrial septum. The left ventricular localization of a myxoma is very rare, usually arising from the interventricular septum close to the left ventricular outflow tract [1-3], the mitral valve [4,5], the left ventricular wall [6] and extremely rarely the aortic valve [7]. Here we report a case of an aortic valve myxoma undergoing successful resection.

## Case presentation

**Figure 1:**
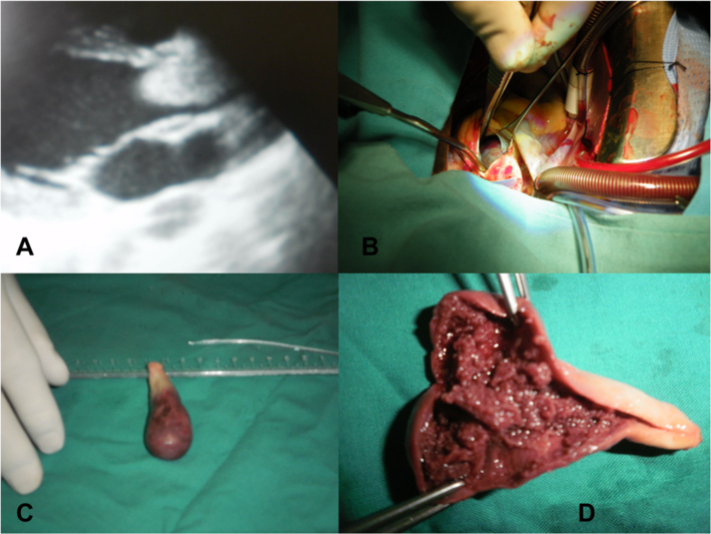
Transthoracic echocardiography, aortotomy, and excision and division of the mass. **(A)** Transthoracic echocardiography revealing a mass at the left ventricular outflow tract. **(B)** Aortotomy was performed and the mass visualized underneath the aortic valve. **(C)** The total excised mass. **(D)** The mass was divided and multiple haemorrhagic areas discovered.

**Figure 2:**
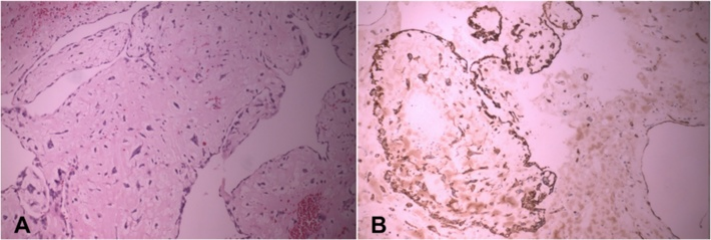
Histopathological examination. **(A)** The myxoma cells with a stellate appearance proliferate in a myxoid background in nests and linear syncytia, which often appear to emanate from vascular structures (H-E, 10X). **(B)** The myxoma cells variably express endothelial cell markers (CD 34, 20×).

A 13-year-old male patient was admitted due to dyspnea and angina. On physical examination, peripheral pulses were present and normal. An early diastolic murmur could be heard in the aortic valve area. The ECG showed a normal sinus rhythm with signs of mild left ventricular hypertrophy. Transesophageal echocardiography revealed a grade II/IV aortic incompetence and left ventricular outflow tract obstruction with a peak gradient of 110 mmHg and a mean gradient of 58 mmHg, and a giant mobile mass originating from the ventricular surface of the aortic valve was identified (Figure 1A). LV function was normal. The other cardiac valves and cavities were free of lesions. The patient underwent urgent surgical excision through a median sternotomy under normothermic cardiopulmonary bypass. The aorta was clamped and an anterior cardioplegic solution was administered. An anterior oblique aortotomy was performed. The inspection of the aortic valve revealed a 65 × 25 mm mass, attached to the ventricular surface of the right and left coronary leaflets (Figure 1B). The mass was gelatinous soft (Figure 1C) with multiple haemorrhagic areas and was totally excised (Figure 1D). The patient underwent standard aortic valve replacement with mechanical prosthesis. Histopathological examination demonstrated a sparse population of round and stellate cells mostly concentrating beneath the surface, with some cells forming solid cords and vascular channels (Figure 2A), surrounded by abundant myxoid stroma (Figure 2B) confirming the diagnosis of a myxoma. Mitosis, pleomorphism and necrosis were all absent. The postoperative course was uneventful. The patient was followed up for 3 years postoperatively with a series of echocardiography control. Non-tumour recurrence was diagnosed.

## Discussion

Myxomas of the left ventricle are very rare. Recently, a thorough literature review demonstrated only 71 cases with a reported left ventricular myxoma usually originating from the interventricular septum [6]. Myxomas of the cardiac valves are very unusual, especially those of the aortic valve. To our knowledge, this is the tenth reported case of an aortic valve myxoma. The first was described as a *post*-*mortem* finding [8]. However, the patient in the present case is the youngest amongst them.

The shape, the extension, the site of attachment, the involvement of valve leaflets and the functional obstruction of the LV outflow tract could promptly and easily be assessed by echocardiography. Transesophageal echocardiography enables the detection of an aortic valve myxoma; however, histopathology remains the gold standard method of diagnosis. Most of the tumours were larger than 1 cm; however, the present case had the greatest mass dimensions amongst the reported cases with an aortic valve myxoma.

Our patient referred dyspnea and angina. The clinical presentations of the other seven reported cases included acute embolic stroke, acute embolic myocardial infarction, acute embolic lower limb ischaemia and aortic stenosis (Table 1). An aortic valve myxoma has been described as arising from both the ventricular aspect [9-11] and the margin of the valve cusps [12]. The right, left and non-coronary leaflets may be affected either together or individually (Table 1). In the other two reported cases [13,14], the right and left cusps were simultaneously affected as in our patient.

**Table 1:** **Clinical presentations of the reported cases and the present case**

**Reference**	**Age**	**Clinical finding**	**Size**	**Complication**	**Associated procedure**	**Location**	**Comorbidity**
Kennedy *et al.* [13]	23	Leg pain	1.5 cm	PVD	AVR	RCC and LCC	None
Watarida *et al.* [9]	58	Heart murmur	1.1 × 1 cm	None	AVR	RCC	HTN
Ramsheyi *et al.* [10]	32	Facial hemiparesis	1 cm	Stroke	AVR	RCC	None
Okamoto *et al.* [11]	61	Endocarditis	1 × 1 cm	None	None	LCC	HTN, DM
Dyk *et al.* [12]	15	Chest pain	4 × 1 cm	STEMI	None	NCC	None
Koyalakonda *et al.* [15]	60	Paroxysmal A-fib	1 × 1 cm	Stroke	None	RCC	A-fib, HTN
Kim *et al.* [16]	72	Shortness of breath	1.5 × 0.8 cm	None	AVR	NCC	HTN, A-fib
Fernandez *et al.* [14]	28	Hemiparesis	1.5 × 0.7 cm	Stroke	AVR	RCC and LCC	Epilepsy
Javed *et al.* [7]	81	Leg pain	1.8 × 1.2 cm	AMI	CABG	LCC	HTN, AA
This study	13	Dyspnea and angina	60 × 22 mm	None	AVR	RCC and LCC	None

AVR, aortic valve replacement; CABG, coronary artery bypass grafting; DM, diabetes mellitus; HTN, hypertension; LCC, left coronary cusp; NCC, non-coronary cusp; RCC, right coronary cusp; PVD, peripheral vascular disease; AMI, acute myocardial infarction; STEMI, ST segment elevation myocardial infarction.

Differential diagnosis of an aortic valve myxoma includes vegetations, papillary fibroelastoma and Lambl’s excrescences. Microscopic and immunohistochemical characteristics allow the distinction between these entities. As we have observed, aortic valve myxomas are a potential source of emboli; therefore, surgical removal should be indicated as soon as the diagnosis is confirmed. Surgical excision should include not only the tumour but also the implantation site to minimize the risk of local recurrence. Tumour resection with conservation of the native valve should be intended, but sometimes due to a big tumour size and/or structural valve degeneration, replacement of the aortic valve may become necessary as in our case. These patients should be followed carefully due to a high probability of distal tumour growth at the site of previous embolization as well as local recurrence of the tumour.

## Conclusions

In conclusion, a myxoma originating from the aortic valve remains a very rare left ventricular localization of such a tumour. Total resection associated with aortic valve replacement seems to offer an excellent outcome.

## Consent

Written informed consent was obtained from the patient’s legal guardian(s) for publication of this case report and any accompanying images. A copy of the written consent is available for review by the Editor-in-Chief of this journal.
